# Association between serum complement C3 and polycystic ovary syndrome: a systematic review and meta-analysis

**DOI:** 10.3389/fimmu.2026.1885980

**Published:** 2026-08-21

**Authors:** Cunling Zhang, Zhuo Wei, Meiyi Xu, Dan Wu, Wen Li, Yu Zhang

**Affiliations:** 1 Tianjin Institute of Gynecology Obstetrics, Tianjin Central Hospital of Gynecology Obstetrics, Tianjin, China; 2 Tianjin Key Laboratory of Human Development and Reproductive Regulation, Tianjin Central Hospital of Obstetrics Gynecology, Tianjin, China; 3 Flagship Project for the Integrated Traditional Chinese and Western Medicine, Gynecology Department at Tianjin Central Hospital of Gynecology Obstetrics, Tianjin, China

**Keywords:** C3, low-grade chronic inflammation, meta-analysis, PCOS, systematic review

## Abstract

**Background:**

Complement C3 is a key effector molecule of the innate complement system, which participates in inflammatory responses, immune regulation and the maintenance of metabolic homeostasis. Consistency and evidence grade regarding the association between serum C3 levels and polycystic ovary syndrome (PCOS) remain controversial. This systematic meta-analysis aims to assess changes of C3 levels in PCOS patients.

**Method:**

We systematically searched PubMed, Scopus, Web of Science, Cochrane Central Register of Controlled Trials (CENTRAL), China National Knowledge Infrastructure (CNKI), VIP Chinese Science and Technology Journal Database (VIP) and Wanfang Database for studies concerning PCOS and C3, from database inception to December 31, 2025. Literature screening, data extraction and risk of bias assessment using the Newcastle-Ottawa Scale (NOS) were independently performed by two investigators. Sensitivity analysis was performed by sequentially omitting each individual study to verify the robustness of the pooled results. Funnel plots were adopted for preliminary evaluation of publication bias. Subgroup analysis was used to explore potential effect modifiers. All statistical analyses were conducted using R software (version 4.6.0) to guarantee the scientific validity and reliability of the findings.

**Results:**

A total of 6 studies meeting the predefined inclusion criteria were included, enrolling 915 individuals. C3 levels were higher in PCOS patients than controls (SMD = 0.73, 95% CI: 0.54–0.93), with moderate heterogeneity (I²=47.1%, P = 0.092). No significant difference was observed between subgroups. Sensitivity analysis confirmed the robustness of the overall association.

**Conclusion:**

This meta-analysis suggests that circulating C3 levels are elevated in PCOS. However, due to the observational design and limited evidence, these findings are exploratory. Prospective studies are needed to validate this association.

**Systematic review registration:**

https://www.crd.york.ac.uk/PROSPERO/, identifier CRD420251121390.

## 1 Introduction

Polycystic ovary syndrome (PCOS) is a common and complex endocrine disorder impacting women of reproductive age, with a global prevalence from 4% to 21%, which depends on the diagnostic criteria applied and the population (1). PCOS exhibits significant heterogeneity, presenting a wide array of clinical symptoms and diverse phenotypic profiles that alter across adolescence and adulthood, as well as differ among distinct racial and ethnic groups (2, 3). The current diagnostic criteria, largely based on expert consensus, mainly focus on the reproductive aspects of PCOS. However, accumulating evidence indicates that low-grade chronic inflammation serves as the core pathophysiological mechanism mediating endocrine, metabolic and reproductive dysfunction in PCOS (4, 5). Consistent with this pathological paradigm, patients with PCOS present elevated levels of leukocytes, CRP, IL-6, IL-18, TNF-α, MCP-1, MIP-1α and other pro-inflammatory mediators (6, 7). In contrast, anti-inflammatory cytokines including IL-10, IL-17E, IL-27, IL-35, IL-37, TGF-β, omentin-1 and secreted frizzled-related protein 5 (SFRP5) are markedly reduced (8).

In the innate immune system that mediates chronic inflammation, the complement system has been proven to serve as a core bridge linking immune surveillance and metabolic dysfunction. The complement cascade acts as a potent amplifier of inflammation through its cleavage products C3a and C5a and the membrane attack complex. Specifically, activation of complement component C3 generates bioactive cleavage products that contribute to chronic metabolic inflammation, insulin resistance and ovarian dysfunction, suggesting its involvement in the pathogenic processes of PCOS (9, 10).

Notably, existing studies on C3 concentrations in PCOS are limited by small sample sizes, inadequate stratification by ethnicity or BMI, and high between-study heterogeneity; no quantitative synthesis has been performed to date. We therefore conducted the first systematic review and meta-analysis to: ① quantify the difference in circulating C3 levels between women with PCOS and control subjects (primary outcome); and ②; explore the modifying effects of region, detection method, BMI, and age.

## 2 Materials and methods

### 2.1 Registration

This systematic review was conducted in accordance with the Preferred Reporting Items for Systematic Reviews and Meta-Analyses 2020 (PRISMA 2020) guidelines (11). The study protocol was registered on the International Prospective Register of Systematic Reviews (PROSPERO), with the registration number CRD420251121390. The quality of evidence was evaluated using the Grading of Recommendations Assessment, Development and Evaluations (GRADE) framework.

### 2.2 Literature search strategy

#### 2.2.1 Databases and time scope

A comprehensive search was conducted across seven electronic databases: PubMed, Scopus, Web of Science, Cochrane Central Register of Controlled Trials (CENTRAL), China National Knowledge Infrastructure (CNKI), VIP Chinese Science and Technology Journal Database (VIP), and Wanfang Database. Besides, we also searched 1 clinical trial register platforms: ClinicalTrials.gov (www.ClinicalTrials.gov/) for in-progress trials with unpublished data. Articles were included from the inception of each database up to December 31, 2025.

To ensure timeliness, we updated our literature search prior to submission. The supplementary search covered January 1, 2026 to May 20, 2026, using the same strategy as the original. No additional eligible studies were identified. Thus, December 31, 2025 was retained as the final cutoff date.

#### 2.2.2 Search terms

A combination of subject headings and free-text terms was employed and the formal search strategy was finalized on the basis of multiple preliminary searches. Search strategy (PubMed example) ((“Polycystic Ovary Syndrome”[Mesh] OR “polycystic ovary syndrome”[tiab] OR “polycystic ovary disease”[tiab] OR “polycystic ovarian syndrome”[tiab] OR pcos[tiab]) AND (“Complement C3”[Mesh] OR “complement c3”[tiab] OR “complement component 3”[tiab] OR “complement component iii”[tiab] OR “c iii”[tiab] OR c3[tiab])). The detailed search strategy is presented in **Supplementary Table 1**.

### 2.3 Literature inclusion and exclusion criteria

#### 2.3.1 Inclusion criteria

Studies included in the meta-analysis must meet all of the following inclusion criteria based on the PICOS principles (12). 1) Population: For the case group, adult women (≥18 years old or referred as adult women in the article) diagnosed with PCOS were included, with any form of PCOS diagnosis (Rotterdam criteria, NIH criteria, AE-PCOS criteria, self-reported diagnosis, International Classification of Diseases [ICD] codes); 2) Intervention/Exposure: PCOS status. 3) Comparison: Healthy women without PCOS, who had no hyperandrogenemia, ovulatory dysfunction or polycystic ovarian morphology, and were not complicated with autoimmune diseases, chronic inflammatory diseases, liver and kidney diseases, or any other disorders affecting serum complement C3 levels. 4) Outcome measure: Serum C3 concentration (including mean ± standard deviation, median, and other combinable statistical measures. 5) Study design: Observational studies (cross-sectional, case-control, cohort baseline comparisons).

#### 2.3.2 Exclusion criteria

Exclusion criteria: 1) For the PCOS group: individuals with PCOS-related confounding conditions (e.g., Cushing’s syndrome, thyroid dysfunction, ovarian tumors) were excluded. Pregnant individuals and patients with systemic diseases involving the kidney, liver, heart, or other systemic disorders were further excluded. 2) Duplicate publications, case reports, conference abstracts, review articles, editorials, animal studies, *in vitro* experimental studies, studies without a control group, and non-peer-reviewed reports. 3) Studies with insufficient raw data that could not be extracted for quantitative analysis.

### 2.4 Data extraction and quality assessment

#### 2.4.1 Data extraction

Two reviewers independently screened the literature and extract data according to the predefined search strategy, with cross-checking of the results. An Excel spreadsheet was used to extract data, including first author, year of publication, study design, sample size, diagnostic criteria for PCOS, country, detection methods for C3 measurement, BMI, age and reported outcomes (mean ± SD). Any disagreements will be resolved through discussion; if no consensus is reached, a third reviewer will be consulted to arbitrate.

#### 2.4.2 Quality assessment

The methodological quality of the included case-control studies was independently assessed by two reviewers using the Newcastle-Ottawa Scale (NOS), which evaluates three domains: selection of cases and controls, comparability, and exposure assessment. Any discrepancies were resolved by consensus or consultation with a third reviewer. The NOS scores ranged from 5 to 6 (mean: 5.5), categorizing all studies as moderate quality based on the predefined threshold (scores of 5–6 for moderate quality). No study was rated as high quality (≥7) or low quality (≤4).

#### 2.4.3 Grading the quality of evidence

We assessed the certainty of evidence using the Grading of Recommendations Assessment, Development and Evaluation (GRADE) framework. Evidence quality was evaluated based on five predefined core GRADE domains: risk of bias, inconsistency, indirectness, imprecision, and other additional considerations. The certainty of evidence was classified into four hierarchical grades: high, moderate, low, and very low. For the included observational studies (cross sectional and case control designs), the baseline certainty of evidence was initially rated as low per official GRADE guidelines. The final Summary of Findings (SoF) table was generated using the GRADEpro Guideline Development Tool (GDT) software.

### 2.5 Statistical analysis

All statistical analyses were performed using R software (version 4.6.0). Since the units of circulating C3 concentrations varied across studies, we used the standardized mean difference (SMD) as the effect size metric. Both common-effect and random-effects models were fitted. The between-study variance (τ²) in the random-effects model was estimated using the DerSimonian–Laird (DL) method. Between-study heterogeneity was assessed using the I² statistic and the Q-test. Given the moderate heterogeneity detected (I²=47.1%), the pooled estimate from the random-effects model was reported as the primary finding. For sensitivity purposes, the Hartung–Knapp–Sidik–Jonkman (HKSJ) adjustment was applied to calculate modified 95% confidence intervals to accommodate the small number of included studies (k = 6). Subgroup analyses were conducted to explore potential sources of heterogeneity. Sensitivity analyses were performed using two complementary approaches: (1) alternative τ² estimation methods (DL and restricted maximum likelihood (REML)) alongside the common-effect model; and (2) leave-one-out analysis, in which each study was sequentially omitted to assess the influence of individual studies on the pooled estimate. Funnel plots were visually inspected to evaluate potential publication bias. Of note, this assessment should be interpreted cautiously given the limited number of included studies.

## 3 Results

### 3.1 Literature screening results

**Figure 1:**
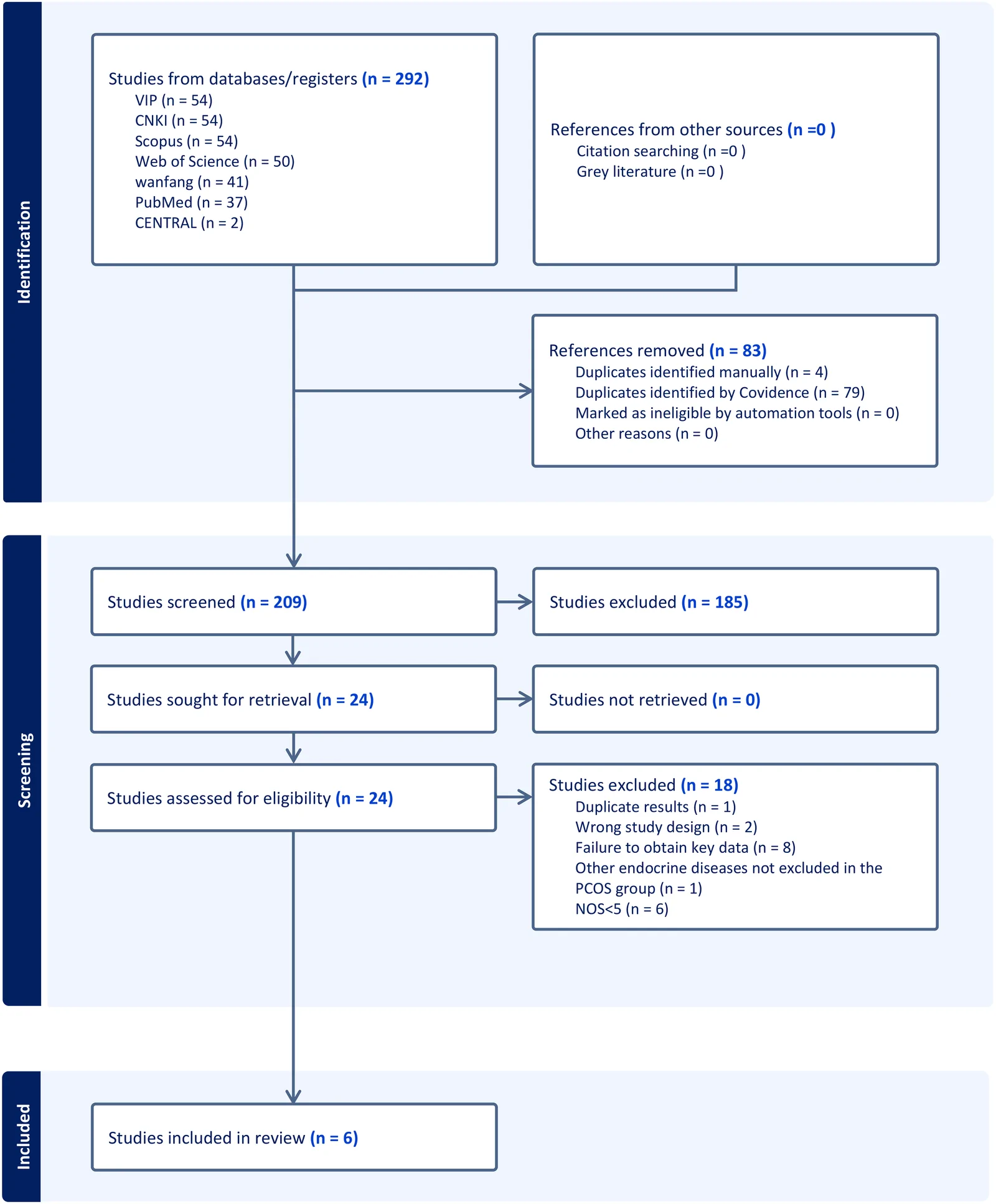
PRISMA flow diagram of literature screening.

We followed the PRISMA 2020 guidelines for study selection. A total of 292 records were identified from PubMed (n=37), Web of Science (n=50), Scopus (n=54), Cochrane CENTRAL (n=2), and Chinese databases (n=149, including CNKI, Wanfang, and VIP), with no additional records identified through other sources. After removing 83 duplicates using Covidence software and manual cross-checking, 209 records remained. During initial title–abstract evaluation, 185 records were excluded, and 24 studies proceeded to full-text assessment. In the full-text screening, 18 studies were excluded for the following reasons: duplicate results (n=1), wrong study design (n=2), failure to obtain key data (n=8), other endocrine diseases not excluded in the PCOS group (n=1), NOS<5(n=6) (**Supplementary Table 2**). Ultimately, 6 eligible studies were included in the final meta-analysis. The entire literature screening procedure is presented in the PRISMA flow diagram (**Figure 1**).

### 3.2 Basic characteristics of included studies

Six case-control studies published between 2007 and 2021 were included, enrolling 915 participants (464 PCOS patients and 451 controls). All studies adopted the 2003 Rotterdam criteria for PCOS diagnosis. Geographically, the included studies covered three regions: Western Europe, the Middle East, and East Asia (**Table 1**). All studies received NOS scores ≥5, indicating moderate methodological quality (**Table 2**). Detailed information on medication use is presented in **Supplementary Table 3**.

**Table 1:** Basic characteristics of the included literature. PCOS, Polycystic Ovary Syndrome; C3, complement. .

Study	Year	Study design	PCOS criteria	Sample size (PCOS/Control)	Country	Region	PCOS BMI (mean ± SD) (kg/m^2^)	Control BMI (mean ± SD) (kg/m^2^)	PCOS age (mean ± SD) (year)	Control age (mean ± SD) (year)	PCOS C3 (mean ± SD) mg/dl	Control C3 (mean ± SD) mg/dl	Detection method
Oktenli (13)	2007	Case-Control	Rotterdam criteria	18/20	Turkey	Middle East	22.05 ± 1.11	21.6 ± 1.52	20.89 ± 1.28	21.1 ± 1.07	140 ± 44	100 ± 16	rate nephelometry
Yuwen Wu (14)	2008	Case-Control	Rotterdam criteria	34/41	China	East Asia	24.62 ± 4.66	23.49 ± 4.22	24 ± 6	26 ± 5	202 ± 103	182 ± 136	turbidometry
Lewis (39)	2021	Case-Control	Rotterdam criteria	84/95	The United Kingdom	Western Europe	33.3 ± 7.8	27.6 ± 6.3	29.8 ± 6.7	32.6 ± 7.9	167 ± 39	140 ± 36	nephelometry
Q.Cheng (40)	2013	Case-Control	Rotterdam criteria	96/63	China	East Asia	23.14 ± 4.21	21.12 ± 2.18	25.48 ± 4.33	25.41 ± 2.47	133 ± 34	115 ± 25	rate nephelometry
Shumin Yang (41)	2011	Case-Control	Rotterdam criteria	133/116	China	East Asia	23.53 ± 4.39	20.96 ± 2.16	25.47 ± 4.41	25.92 ± 2.67	137 ± 34	110 ± 22	rate nephelometry
Wenwen He (42)	2016	Case-Control	Rotterdam criteria	99/116	China	East Asia	23.23 ± 4.3	20.96 ± 2.61	25.62 ± 4.38	25.92 ± 2.67	133 ± 34	110 ± 22	rate nephelometry

**Table 2:** The Newcastle-Ottawa scale served to assess the risk of bias in the included studies.

Study, year	Selection				Comparability		Exposure			Summary	
	Adequate case definition	Case representativeness	Control selection	Control definition	Matched in design	Adjusted in analyses	Exposure ascertainment	Same method of ascertainment for cases and controls	non-response rate for both groups	Points	Risk of bias
Oktenli,2007 (13)	–	–	–	*	*	*	*	*	–	5	Moderate
Yuwen Wu, 2008 (14)	–	*	–	*	*	–	*	*	–	6	Moderate
Lewis, 2021 (39)	–	–	*	–	*	*	*	*	–	5	Moderate
Q. Cheng, 2013 (40)	–	*	*	*	*	–	*	*	–	6	Moderate
Shumin Yang 2011 (41)	–	*	–	*	*	*	*	*	–	6	Moderate
Wenwen He, 2016 (42)	–	*	–	*	*	–	*	*	–	5	Moderate

The symbol * indicates that the corresponding item received 1 star in the NOS scoring system.

### 3.3 Meta-analysis of C3 level between PCOS and controls

**Figure 2:**
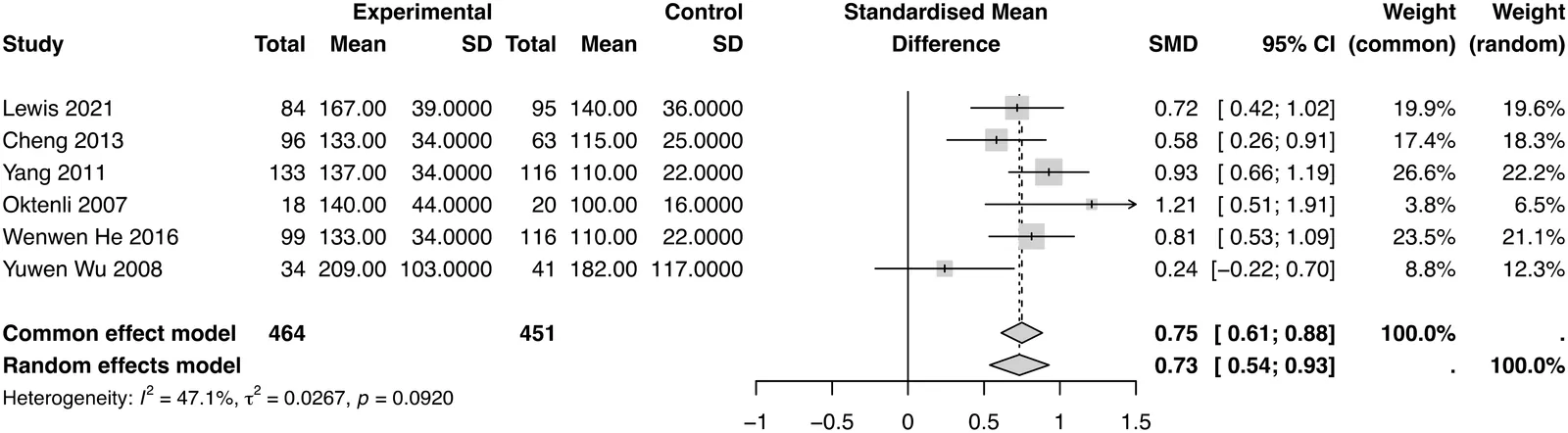
Forest plot summarizing the association between serum C3 levels and PCOS.

As illustrated in **Figure 2**, a total of 6 studies were ultimately enrolled in the meta-analysis. Moderate between-study heterogeneity was detected (I^2^ = 47.1%, τ²=0.026, P = 0.092), so the random-effects model was selected as the primary analytical method. The pooled estimate showed that serum C3 levels were higher in the PCOS group than in the control group (pooled SMD = 0.73, 95% CI: 0.54-0.93). The common-effect model produced consistent outcomes (SMD = 0.75, 95% CI: 0.61-0.88), supporting the robustness of the pooled effect.

### 3.4 Subgroup analysis

**Figure 3:**
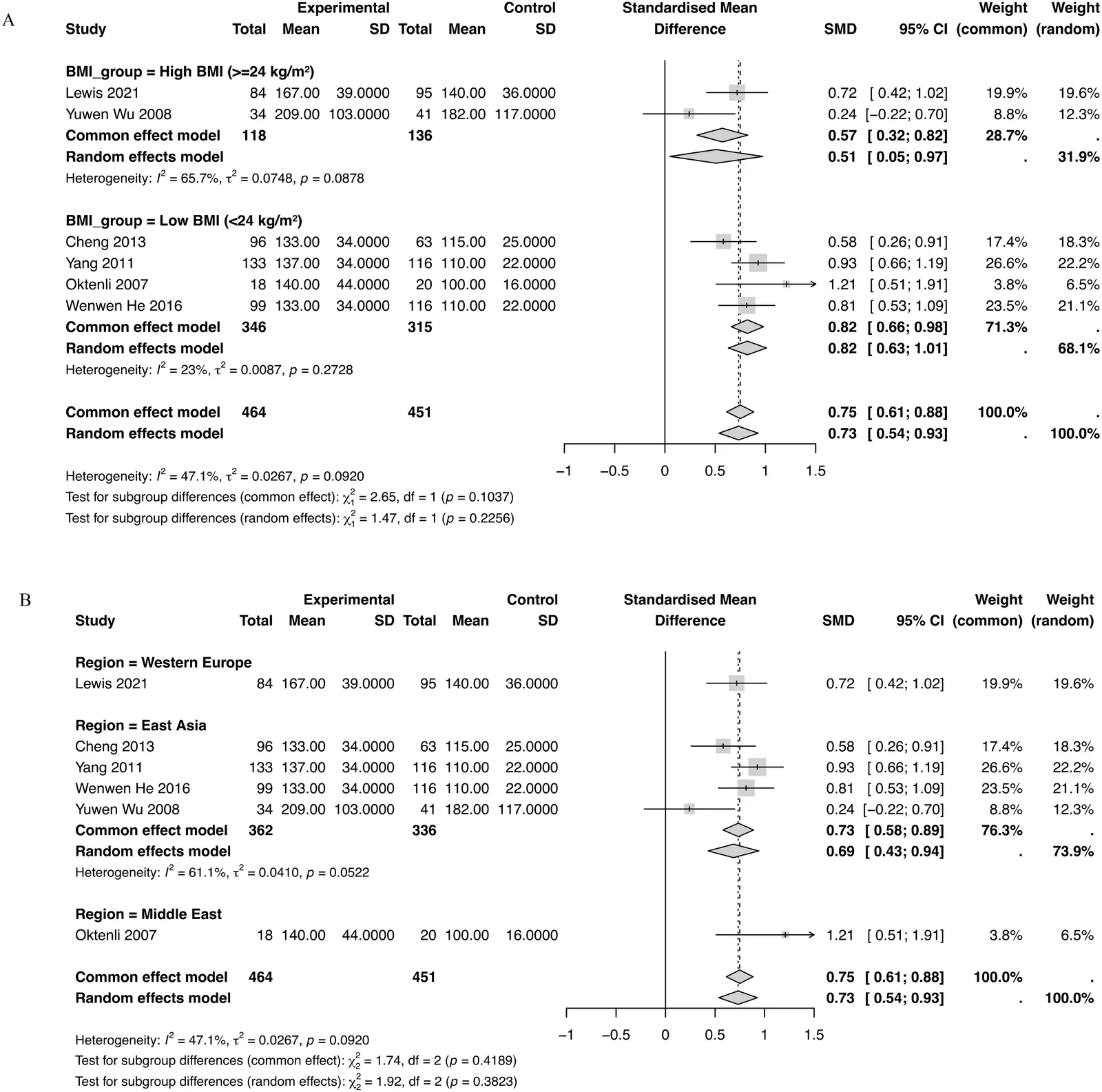
Subgroup analysis forest plot stratified by BMI **(A)** and region **(B)**.

**Figure 4:**
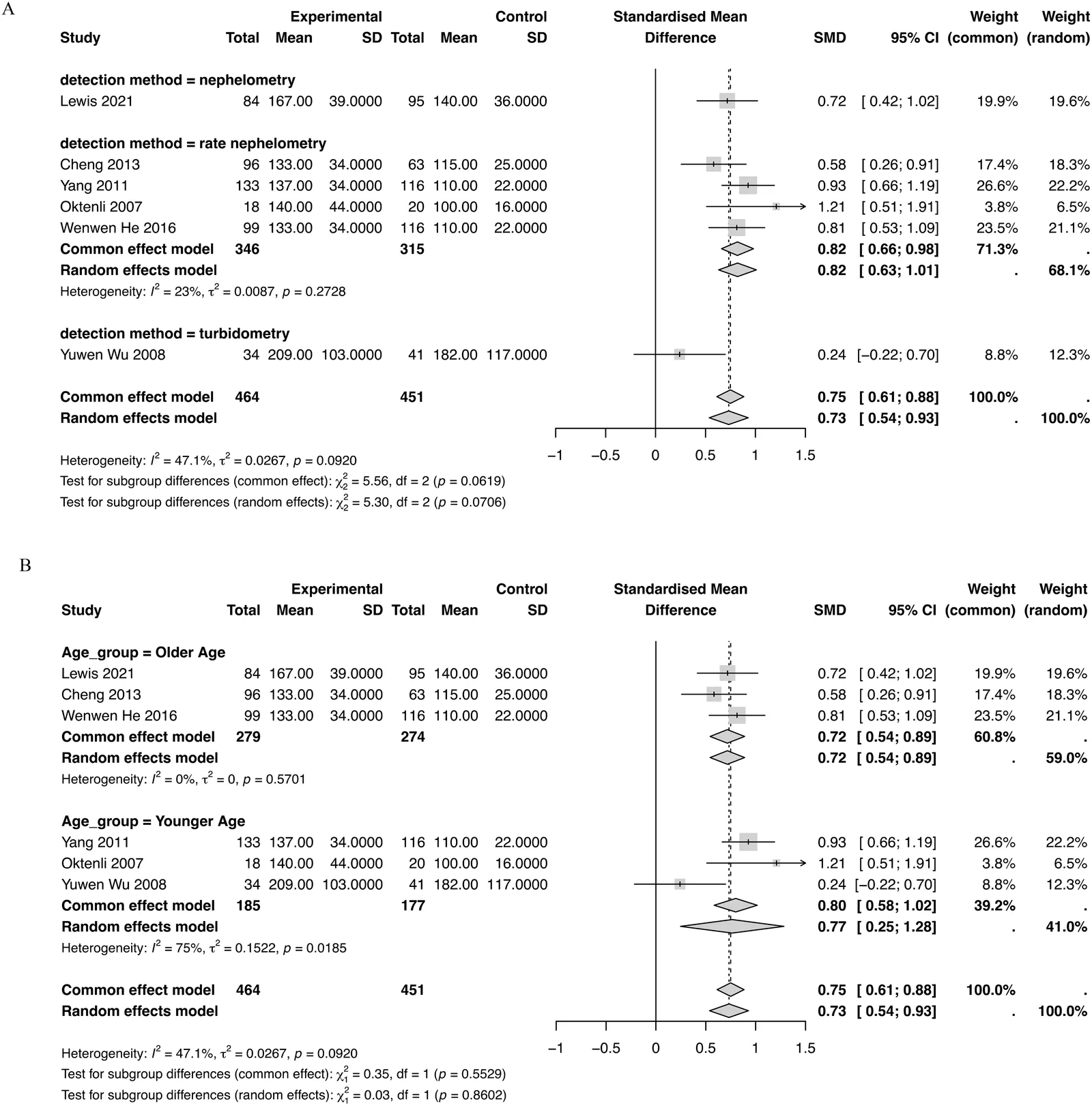
Subgroup analysis forest plot stratified by detection method **(A)** and age **(B)**.

Subgroup analyses were performed to investigate the moderate heterogeneity (I²=47.1%, P = 0.092), stratified by BMI, geographic region, detection method, and age (**Figures 3**, **4**). Using the Asian overweight threshold of 24 kg/m², the low-BMI subgroup (4 studies) yielded a pooled SMD of 0.82 (95% CI: 0.63-1.01, I²=23%), compared with 0.51 (95% CI: 0.05-0.97, I² = 65.7%) in the high-BMI subgroup (2studies). The difference between subgroups was not statistically significant (**Figure 3A**). When grouped by geographic region, pooled estimates were similar across East Asian, Western European, and Middle Eastern cohorts, with no evidence of subgroup differences (**Figure 3B**). For detection method, nephelometry-based studies yielded a pooled SMD of 0.82 (95% CI: 0.63-1.01, I²=23%), whereas the single turbidimetric study reported 0.24 (95% CI: −0.22 to 0.70). The subgroup difference was borderline but not significant (P = 0.071) (**Figure 4A**). Age-based stratification showed nearly identical estimates between younger and older subgroups (**Figure 4B**). Overall, none of the examined factors clearly accounted for the observed heterogeneity.

### 3.5 Sensitivity analysis

**Figure 5:**
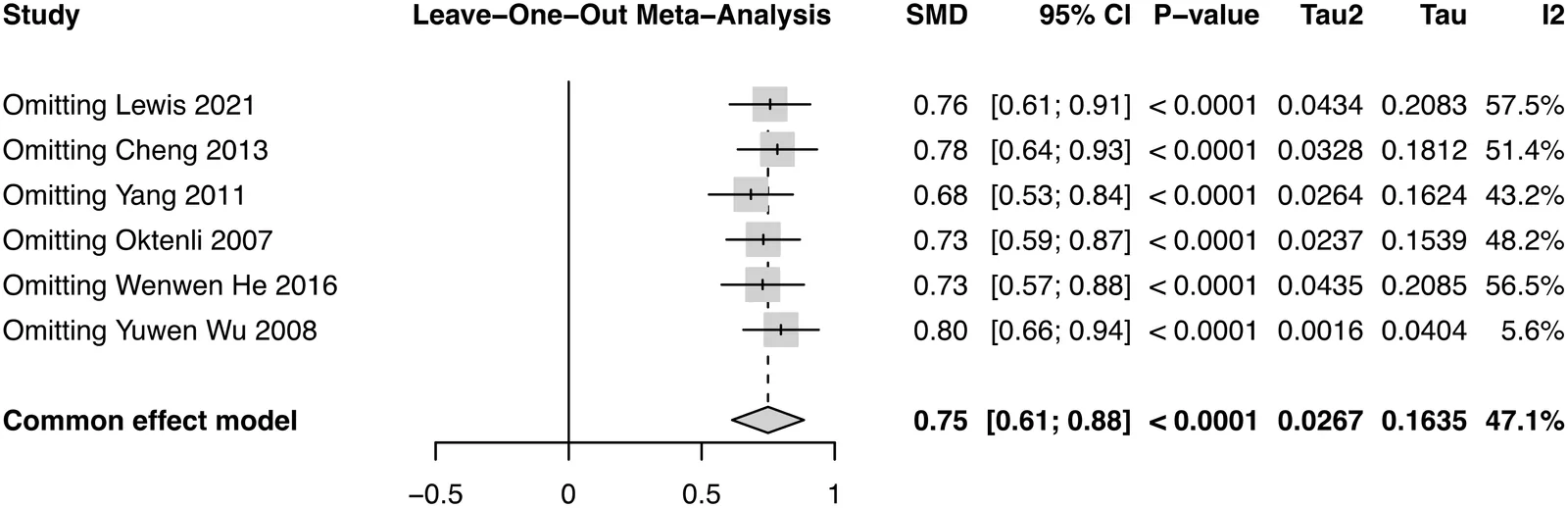
Leave-one-out sensitivity analysis.

Sensitivity analysis was conducted to evaluate the stability of the pooled effect size. Sequential omission of each individual study showed that the pooled SMD ranged from 0.68 to 0.80 (**Figure 5**). In all cases, the 95% CI did not cross the null line, and all P-values remained below 0.0001, with no reversal in the direction of effect. Excluding the small-sample, high-effect study (13) produced a pooled estimate almost identical to the original. Removing the only study whose confidence interval contained zero (14), led to a slight increase in the pooled effect and a marked drop in heterogeneity. Taken together, these findings suggest that the pooled result is stable and the conclusions are robust. We further tested alternative meta-analytic models with different τ² estimators. The pooled SMD ranged between 0.733 and 0.749, and all 95% CI excluded zero. Consistent results were observed across DL, REML, ML random-effects models, common-effect model and Hartung–Knapp–Sidik–Jonkman (HKSJ) correction (**Supplementary Table 4**), supporting the reliability of the primary analysis.

### 3.6 Publication bias

**Figure 6:**
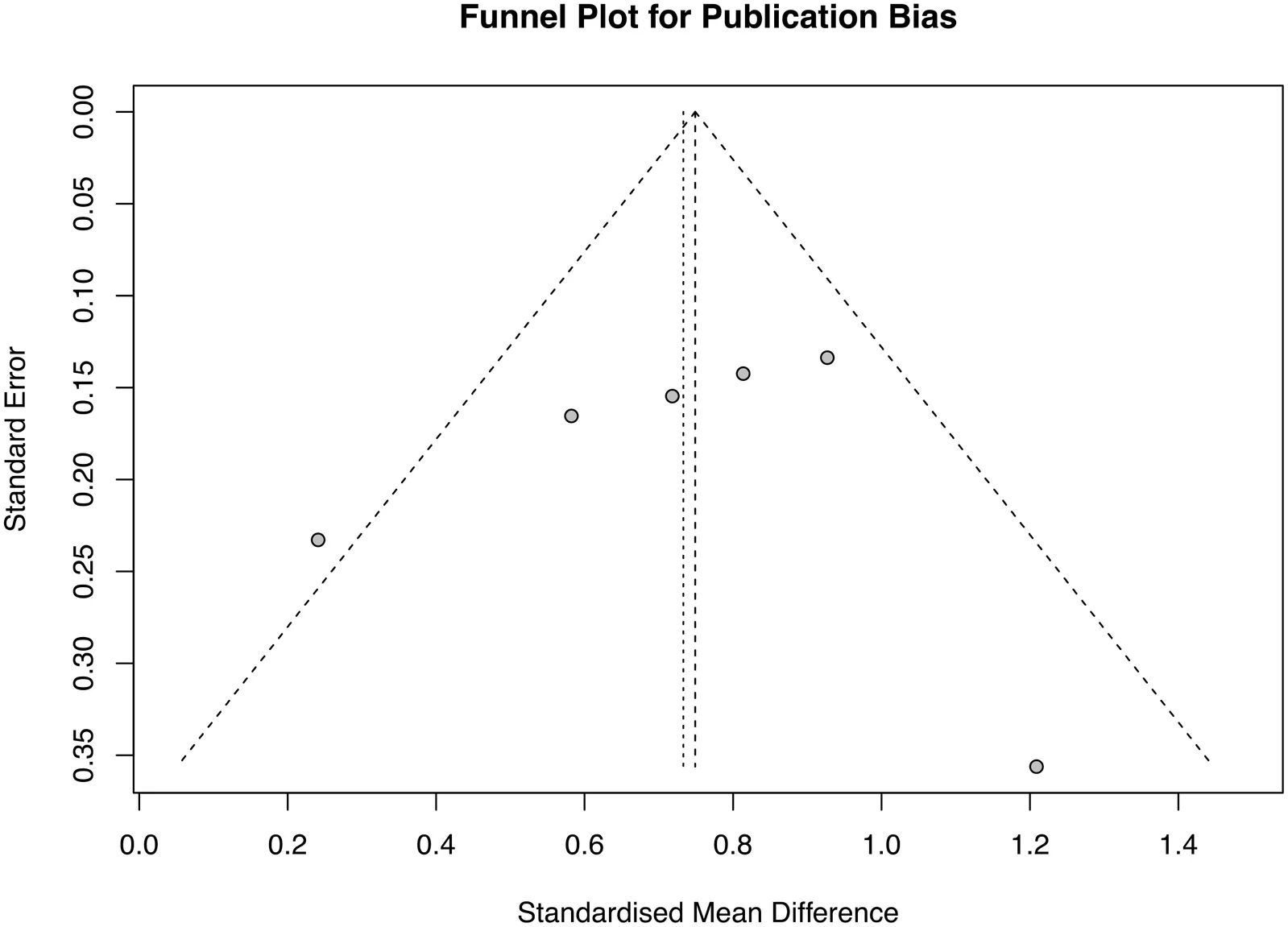
Funnel plot for assessment of publication bias in the meta-analysis of serum C3 levels between PCOS patients and healthy controls. The vertical dashed line represents the pooled effect estimate, and the diagonal dotted lines represent the 95% confidence interval around the pooled effect.

Visual inspection of the funnel plot revealed mild asymmetry, with fewer small studies reporting low effect estimates (**Figure 6**). Formal statistical tests for publication bias were not performed, as such tests are underpowered when fewer than 10 studies are included. Given the moderate between-study heterogeneity (I^2^ = 47.1%), the observed asymmetry may partly reflect inter-study variation rather than publication bias alone.

### 3.7 GRADE evidence quality assessment

We assessed the certainty of evidence using the GRADE framework. The initial evidence quality was rated low, as all included studies were observational case–control designs. We downgraded two levels for serious risk of bias and serious imprecision. Moderate heterogeneity was not considered serious inconsistency and did not warrant further downgrading. Although the large effect size raised the possibility of upgrading, the overall certainty remained very low. Asymmetry in the funnel plot raised concerns about potential publication bias, further supporting the very low certainty rating and limiting causal inferences (**Supplementary Table 5**).

## 4 Discussion

This meta-analysis included six case–control studies with a total of 915 participants to compare serum complement C3 levels between women with PCOS and healthy controls. The pooled results showed that C3 concentrations were higher in women with PCOS (SMD = 0.73, 95% CI: 0.54–0.93). Subgroup analyses were performed using the BMI cutoff for overweight in Asian populations. The association remained robust among normal-weight women with PCOS (SMD = 0.82, I^2^ = 23%), whereas the effect size was weak and accompanied by substantial between-study heterogeneity in overweight/obese subgroups (SMD = 0.51, I^2^ = 65.7%). This finding suggests a potential modifying effect of BMI on the relationship between PCOS and C3, although the subgroup difference did not reach statistical significance. Given the limited number of studies in the overweight/obese subgroup, the high I² value should be interpreted with caution. Multiple sensitivity analyses confirmed that the overall pooled estimate was not driven by any single study, supporting the stability of our main results. Mild asymmetry was observed on the funnel plot, nevertheless, conclusions regarding publication bias should be interpreted cautiously, given that only six studies were eligible for inclusion (k=6).

PCOS is a complex etiology involving genetic, epigenetic, and environmental factors (15, 16) affecting more than 170 million women worldwide (17). Its core clinical features include menstrual irregularities, hyperandrogenism, ovulatory dysfunction, and infertility, accompanied by metabolic disturbances such as insulin resistance, obesity, and dyslipidemia, which significantly increase the risk of cardiometabolic diseases (17–19). Chronic low-grade inflammation is regarded as a central mediator in the pathophysiology of PCOS (16) and a key feature of its immune dysfunction. Even in the absence of acute infection, patients exhibit persistent, mild systemic inflammation, with markedly elevated circulating levels of inflammatory markers including CRP, IL-6, and TNF-α (20). Adipose tissue, especially visceral fat, serves as a major source of these proinflammatory mediators (21). Inflammation in PCOS is not just an incidental finding, but is directly driven by the PCOS phenotype or indirectly mediated by obesity and insulin resistance. It also impairs endometrial function by altering the proliferation and apoptosis of endometrial cells, thereby increasing the risk of endometrial hyperplasia and carcinogenesis. Although other mechanisms have been proposed, including hyperandrogenemia, oxidative stress, abnormal vitamin D levels, and insulin resistance (18, 19), the central role of chronic inflammation in the pathogenesis of PCOS is widely recognized.

As a vital component of innate immunity, C3-the core component of the complement system-is abundant in serum. Beyond its pivotal roles in inflammatory regulation and immune clearance, C3 also plays critical functions in metabolic homeostasis. C3 affects insulin secretion and adipocyte maturation, serving as an important molecule regulator of metabolic organs and the progression of metabolic diseases (22). Multiple studies have confirmed that abnormal activation of the complement system can aggravate inflammatory responses in obesity, insulin resistance, type 2 diabetes, non-alcoholic fatty liver disease, and cardiovascular diseases (23–26), all of which are closely associated with PCOS (27). Combined single-cell transcriptomic and *in vitro* cellular experiments confirmed that aberrant overexpression of C3 drives excessive complement system activation and triggers chronic low-grade inflammation. This process subsequently impairs granulosa cell function, follicular development and maturation, and leading to typical pathological features of PCOS such as hyperandrogenism, insulin resistance, and ovulatory dysfunction (10). Although direct clinical and mechanistic studies focusing on the association between PCOS and complement C3 remain limited, potential associations can be inferred from their respective roles in metabolism and inflammation (28–30).

Our core finding is consistent with previous reports. Abdul-Ameer et al. (31) confirmed higher C3 levels in PCOS cohorts. In a large-cohort investigation, Butler et al. (32) reported significant increases in alternative pathway proteins (C3, properdin, and factor B), suggesting complement activation. Together, these independent findings from diverse populations and assays support that elevated complement C3 is a consistent feature in PCOS.

Moderate between-study heterogeneity was observed (I^2^ = 47.1%). We performed predefined subgroup and sensitivity analyses to explore its potential sources. No statistically significant subgroup differences were identified, which indicates that the selected stratification variables could not fully account for the heterogeneity. Exploratory trends were observed for two stratification factors: BMI and detection method.

A larger effect size in the low-BMI subgroup than in the high-BMI subgroup was observed in subgroup analysis, although the interaction was not statistically significant. This can be explained by increased circulating C3 synthesized and secreted by adipose tissue of individuals dilutes the effect of C3 elevation driven by PCOS itself (33–35). Consistently, Snyder et al. (36) did not observe a significant difference of C3 among PCOS and control individuals exhibited mean BMIs of 31.5 kg/m² and 28.6 kg/m² respectively. And Butler et al. (37, 38) detected marked upregulation of alternative complement pathway proteins in non-obese PCOS patients but not obese PCOS with BMI matched control participants. In summary, consistent with our BMI subgroup analysis, this implies that differences in population BMI may partly explain inter-study variation.

A borderline trend was observed by detection modality in subgroup analysis (P = 0.071). However, with only one study employing turbidimetry, this finding must be interpreted with considerable caution. Notably, the same study (14)was a major driver of between-study heterogeneity in sensitivity analysis, yet its exclusion did not materially alter the overall estimate. This supports the robustness of our main findings. Collectively, the factors examined accounted for only part of the observed heterogeneity, with residual variation likely reflecting unmeasured inter-study differences. This residual variation may stem from inconsistently reported clinical and methodological variables across primary studies, including divergent PCOS phenotypes, severity of insulin resistance, medication history, blood sampling conditions, and laboratory operating protocols. Furthermore, all subgroup analyses relied on aggregate study-level data rather than individual participant data. Accordingly, these group-level trends cannot be directly extrapolated to individual patients. Well-powered primary studies with standardized laboratory protocols and comprehensive clinical reporting are still needed to validate and expand on the present observations.

Several limitations should be considered when interpreting the present findings. First, all included studies adopted a case–control design, which can only reveal cross-sectional associations and cannot establish a temporal or causal relationship between elevated complement C3 and PCOS. Second, most of the original studies had moderate NOS scores, indicating a moderate overall risk of bias, and many did not adequately report important confounders such as insulin resistance, preventing further adjustment in our analysis. Third, the final meta-analysis included only six studies, yielding a limited total sample size and insufficient statistical power. After stratification, some subgroups contained very few studies, making the results susceptible to random error and requiring conservative interpretation. Fourth, formal publication bias tests were not feasible due to the small number of included studies. We therefore relied on visual inspection of the funnel plot for qualitative assessment. Fifth, the study populations were predominantly from East Asia and Europe, with mean ages between 21 and 30 years. Whether these findings can be extended to other ethnic groups, adolescents, or middle-aged and older women with PCOS remains uncertain. Finally, according to the GRADE framework, the overall certainty of evidence was rated as very low, mainly due to the risk of bias in the original studies and the substantial imprecision in the pooled effect estimates.

Despite these limitations, the analytical process of this study strictly followed current methodological guidelines for meta-analyses. All subgroup stratifications were pre-specified in the protocol and aimed at exploring potential sources of heterogeneity rather than drawing definitive clinical conclusions. Thus, the subgroup findings should be viewed as exploratory. To assess the robustness of the pooled estimates, we performed leave-one-out sensitivity analyses and used different τ² estimators in random-effects models. The direction of the pooled effect remained stable across all models, indicating that the core conclusion was not dominated by any single study or model choice. Given the limited number of included studies (k=6), quantitative publication bias tests were not performed. We instead relied on funnel plot inspection for qualitative assessment, to avoid misleading results from underpowered statistical tests. Importantly, statistical robustness does not eliminate the inherent confounding or causal limitations of observational studies. Statistical stability should not be equated with clinical applicability. Collectively, this study represents a preliminary mechanistic exploration of the association between PCOS and complement C3. Future large-scale prospective cohort studies with standardized assays and detailed individual-level data collection are warranted to further validate these findings.

## 5 Conclusion

This meta-analysis suggests that serum complement C3 levels are elevated in women with PCOS compared with healthy controls, with a more consistent effect observed in normal-weight individuals. However, due to the observational study designs and the very low certainty of evidence (GRADE), these findings should be considered exploratory and do not currently support the clinical use of complement C3. Large-scale prospective studies are needed to further validate the association between PCOS and C3.

## Data Availability

The original contributions presented in the study are included in the article/**Supplementary Material**. Further inquiries can be directed to the corresponding authors.
