# Supplementary material for: Association between serum complement C3 and polycystic ovary syndrome: a systematic review and meta-analysis
Source: Front Immunol. 2026 Aug 21;17:1885980. doi: 10.3389/fimmu.2026.1885980 (PMC13541940; doi:10.3389/fimmu.2026.1885980)
Supplement: Supplementary file 1 [file Table1.docx]

Supplementary Material

| **Supplementary Materials Table S1. Search strategy** | | | |
| --- | --- | --- | --- |
| Database | Search strategy | Results | Time |
| Scopus | ( TITLE-ABS-KEY ( "polycystic ovary syndrome" OR pcos OR "polycystic ovary disease" OR "polycystic ovarian syndrome" ) AND TITLE-ABS-KEY ( "complement c3" OR c3 OR "complement component 3" OR "complement component iii" OR "c iii" ) AND TITLE-ABS-KEY ( observational OR cohort* OR "case-control" OR "case control" OR cross-sectional OR "cross sectional" OR patient* OR wom*n ) ) | 54 | December 31,2025 |
| PubMed | (("Polycystic Ovary Syndrome"[Mesh] OR "polycystic ovary syndrome"[tiab] OR "polycystic ovary disease"[tiab] OR "polycystic ovarian syndrome"[tiab] OR pcos[tiab]) AND ("Complement C3"[Mesh] OR "complement c3"[tiab] OR "complement component 3"[tiab] OR "complement component iii"[tiab] OR "c iii"[tiab] OR c3[tiab])) | 37 | December 31,2025 |
| Cochrane  Library | ("Polycystic Ovary Syndrome" OR PCOS OR "polycystic ovary disease"):ti,ab,kw AND ("Complement C3" OR C3 OR "complement component 3" OR "complement component III" OR "Complement C III" OR "C III"):ti,ab,kw AND (observational OR cohort* OR "case-control" OR "cross-sectional" OR "cross sectional"):ti,ab,kw | 2 | December 31,2025 |
| Web of Science  Core collection | "Polycystic Ovary Syndrome" OR PCOS OR "polycystic ovary disease" (Topic) and "Complement C3" OR C3 OR "complement component 3" OR "complement component III" OR "Complement C III" OR "C III" (Topic) and "observational OR cohort*" OR case-control OR cross-sectional OR "cross sectional" OR patient* OR wom*n | 50 | December 31,2025 |
| CNKI | TKA=('多囊卵巢综合征'+'PCOS'+'多囊性卵巢'+'斯坦-利文撒尔综合征')*('补体C3'+'C3'+'补体第三成分'+'补体因子III')*('观察性'+'队列'+'病例对照'+'横断面'+'现况调查'+'患者') | 54 | December 31,2025 |
| VIP | U="多囊卵巢综合征" OR "PCOS" OR "多囊性卵巢" OR "卵巢多囊" OR "斯坦-利文撒尔综合征" AND U="补体C3" OR "C3" OR "补体第三成分" OR "补体因子III" OR "补体成分3" AND U="观察性" OR "队列" OR "病例对照" OR "横断面" OR "现况调查" OR "患者" | 54 | December 31,2025 |
| Wanfang | (((主题=(\"多囊卵巢综合征\" OR \"PCOS\" OR \"多囊性卵巢\" OR \"斯坦\-利文撒尔综合征\" OR \"卵巢多囊\" OR \"多囊卵巢\")) AND 主题=(\"补体 C3\" OR \"C3\" OR \"补体成分 C3\" OR \"补体因子III\" OR \"CIII\")) AND 主题=(\"观察性\" OR \"队列\" OR \"病例对照\" OR \"横断面\" OR \"现况调查\" )) AND 主题=(\"患者\" OR \"病人\" OR \"女性\") | 41 | December 31,2025 |

**Supplementary Materials Table S2: The Newcastle-Ottawa scale served to assess the risk of bias in the excluded studies due to score<5.**

| Study | Selection | | | | Comparability | | Exposure | | | Summary | |
| --- | --- | --- | --- | --- | --- | --- | --- | --- | --- | --- | --- |
|  | Adequate  Case  definition | Case representativeness | Control selection | Control definition | Matched in design | Adjusted in analyses | Exposure ascertainment | Same method of ascertainment for cases and controls | non-response rate for both groups | Total point | Risk of bias |
| Abdul-Ameer, 2024 | 0 | 0 | 0 | 0 | 1 | 0 | 1 | 1 | 0 | 3 | High |
| Khichar,2021 | 0 | 0 | 0 | 0 | 0 | 0 | 1 | 1 | 0 | 2 | High |
| Al-Kubaisi, 2019 | 0 | 0 | 0 | 0 | 0 | 0 | 1 | 1 | 0 | 2 | High |
| Aslan ,2019 | 0 | 0 | 0 | 0 | 0 | 0 | 1 | 1 | 0 | 2 | High |
| Xiaoling Zhang ,2021 | 0 | 0 | 0 | 1 | 0 | 0 | 1 | 1 | 0 | 3 | High |
| Cheng Luo, 2011 | 0 | 1 | 0 | 0 | 1 | 0 | 1 | 1 | 0 | 4 | High |

**Supplementary Materials Table S3. Treatment Status of Included Studies**

| Study | Specific Criteria | Treatment-Naïve Status |
| --- | --- | --- |
| Wenwen He 2016 | No insulin sensitizers within 3 months; no hormonal medications within 1 month | Confirmed |
| Yuwen Wu 2008 | No hormonal medications within 3 months | Confirmed |
| Oktenli 2007 | No subjects taking any medication at the time of study | Confirmed |
| Yang 2011 | No hormonal medications (including OC) within 1 month; no insulin sensitizers within 3 months | Confirmed |
| Cheng 2013 | No hormonal medications (including OC) within 1 month; no insulin sensitizers within 3 months | Confirmed |
| Lewis 2021 | Not excluded; 22.6% (C1) / 40% (C2) of PCOS and 29.5% (C1) / 35% (C2) of controls using combined oral contraceptive pills | Not confirmed; concurrent contraceptive use reported |

**Supplementary Materials Table S4. Sensitivity analysis using different pooled-effect models.**

| Model \| SMD \| 95% CI \| τ² \| I²; DerSimonian–Laird (DL, primary random-effects) \| 0.733 \| 0.538–0.929 \| 0.0267 \| 47.1%; REML random-effects \| 0.735 \| 0.550–0.919 \| 0.0209 \| 47.1%; ML random-effects \| 0.741 \| 0.586–0.897 \| 0.0075 \| 47.1%; Common-effect model \| 0.749 \| 0.614–0.884 \| NA \| 47.1%; Hartung–Knapp–Sidik–Jonkman (HKSJ) correction (based on DL) \| 0.749 \| 0.516–0.983 \| 0.0269 \| 47.1% The I^2^ is calculated from the Q statistic rather than τ². CI, confidence interval; SMD, standardized mean difference. **Supplementary Materials Table S5. GRADE for quality of evidence profile** | | | | | | | | | | |
| --- | --- | --- | --- | --- | --- | --- | --- | --- | --- | --- |
| Outcomes | Study design | Number of studies  (patients) | Risk of bias | Inconsistency | Indirectness | Imprecision | Publication bias | Summary of findings  Pooled effect (95% CI) | Certainty |  |
| C3 | Observational Studies | 6(915) | Serious^a^ | Not Serious^b^ | Not Serious | Serious^c^ | Serious^d^ | Pooled SMD 0.73 (95% CI 0.54 to 0.93)  Higher in PCOS | ⊕⊝⊝⊝  Very Low |  |
|  | Note: a, all included studies were observational case-control designs, the majority scored ≤6 on the Newcastle-Ottawa Scale, suggesting moderate-to-high risk of bias that may overestimate or underestimate the true association. b, moderate heterogeneity (I² = 47.1%), with substantial overlap of confidence intervals across studies; heterogeneity is within an acceptable range. c, the total sample size (n=915) was relatively small, and the 95% CI was wide, leading to serious imprecision. d, visual inspection of funnel plot suggested asymmetry.  CI, confidence interval; GRADE, Grading of Recommendations Assessment, Development and Evaluation; NOS, Newcastle-Ottawa Scale; PCOS, polycystic ovary syndrome; SMD, standardized mean difference. | | | | | | | | |  |
